# Electrocardiographic Characteristics of Healthy Newborns During the First Postnatal Hour

**DOI:** 10.3390/jcdd13030140

**Published:** 2026-03-17

**Authors:** Duygu Besnili Acar, Erkut Ozturk

**Affiliations:** 1 Neonatology Department, Gaziosmanpasa Research and Training Hospital, 34255 Istanbul, Turkey; 2 Pediatric Cardiology Department, Cam Sakura City Hospital, 34480 Istanbul, Turkey; erkut.ozturk@sbu.edu.tr

**Keywords:** newborn, electrocardiogram, ECG variability, early postnatal period

## Abstract

Background: Few studies have reported electrocardiogram data collected during the neonatal period. The aim of this study was to evaluate electrocardiographic parameters in healthy neonates during the first postnatal hour. Methods: Electrocardiogram samples taken during the first hour of life from newborns born at our hospital were analyzed in this prospective observational study. Demographic data and possible electrocardiogram changes were studied. The results were statistically analyzed. Results: A total of 260 patients were included during the study period. Among these, 50% were male (n = 130), the mean gestational age was 38.1 ± 1.4 weeks, and the mean birth weight was 3.2 ± 1.4 kg. In the electrocardiograms obtained, low atrial rhythm was detected in 0.3% of the patients (n = 1). Right axis deviation was observed in 1.5% of the patients (n = 4), and left axis deviation was observed in 1.2% of the patients (n = 3). An abnormal P-axis was found in one patient (0.3%), and an abnormal QRS-T angle was found in one patient (0.3%). No statistically significant differences were observed in the other parameters. Conclusion: Physiologic electrocardiographic findings can be observed during the first postnatal hour. Further studies are needed to clarify the interpretation of the electrocardiogram findings.

## 1. Introduction

Immediately after birth, with the transition from the fetal period to the neonatal period, certain anatomical and hemodynamic changes occur in the cardiovascular system [1,2]. In the fetus, the placenta acts as a low-resistance vascular bed, and the right ventricle (RV) is the dominant ventricle, generating 60% of the cardiac output. After the umbilical cord is cut and breathing begins, there is a decrease in pulmonary vascular resistance and an increase in systemic vascular resistance as the placenta is removed from the circulation. This results in a shift of the dominant ventricle from the RV to the left ventricle (LV).

The transition from fetal to neonatal circulation is characterized by rapid and dynamic hemodynamic and electrophysiologic changes, including decreasing pulmonary vascular resistance, closure of fetal shunts, shifts in ventricular dominance, and autonomic nervous system maturation. These processes substantially influence ECG parameters such as heart rate, QRS axis, QT/QTc intervals, and T-wave morphology.

Hemodynamic and anatomical changes can be assessed in practice via clinical parameters (heart rate, oxygen saturation, respiratory pattern, and cardiac auscultation) and complementary tests (e.g., echocardiogram, serum lactate, and serum sodium bicarbonate). However, the exact effects of these circulatory changes on electrocardiography (ECG) during the first few days are not well understood. In addition, there are fewer electrocardiographic studies in the neonatal period than in other stages of life [3,4,5,6].

Although electrocardiography is widely used in neonatal practice, normative data specifically reflecting the immediate postnatal transition remain limited. Historically, the most comprehensive pediatric electrocardiographic reference dataset was reported by Davignon et al. [4], and has been widely used in clinical interpretation. However, these recordings were obtained within the first 24 h of life and do not specifically represent electrocardiographic findings during the first postnatal hour, a period characterized by rapid hemodynamic adaptation.

The aim of this study was to describe electrocardiographic parameters recorded in healthy neonates during the first postnatal hour.

## 2. Materials and Methods

This prospective observational study was conducted on neonates born at our hospital between 1 November 2023 and 1 February 2024. Newborns who received resuscitation after birth, those with congenital anomalies, advanced preterm infants, those admitted to the neonatal intensive care unit, and those whose families did not give consent were excluded from the study.

The study was designed in accordance with the Declaration of Helsinki after approval by the local ethics committee. A study form was completed for each case, including maternal demographics, gestational age at birth, mode of delivery, sex, birth weight, physical examination findings, and ECG data.

ECGs were recorded via the Philips Page Writer Trim II device (Philips Medical Systems, Andover, MA, USA) at a paper speed of 25 mm/second, an amplitude of 10 mm/mV and a bandwidth filter of F 60 ~ 0.15–150 Hz with 12 leads. The electrodes were placed with solid adhesive gel on the right and left shoulders, the iliac region and the areas recommended for the V1–V6 leads. The shoulders and iliac regions were chosen instead of the arms and legs to reduce artifacts caused by the natural movements of the newborn and to improve the quality of the ECG signal. In our study, ECG parameters were initially obtained using the automated measurement functions of the ECG device and subsequently reviewed manually to ensure accuracy, particularly for wave morphology and axis determination. All ECG recordings and measurements were evaluated by a pediatric cardiologist experienced in neonatal electrocardiography. As the ECG assessments were performed by a single observer, interobserver variability analysis was not applicable. Given the observational and descriptive nature of the study, formal intraobserver variability analysis was not performed; however, all measurements were conducted using standardized criteria and predefined measurement settings to ensure consistency.

The following parameters were assessed:

- Heart rate (bpm, measured automatically by the device).
- Frontal plane QRS axis (°).
- P-wave amplitude (mm), duration (ms) and PR interval in lead II (ms).
- Q-wave amplitude in leads III, aVF and V5–V6.
- S-wave amplitude in leads aVR, V1–V2 and V4–V6.
- R/S ratio in leads V1 and V6.
- QRS duration, QT and QTc intervals (using Bazett’s formula) in lead II (ms).
- T-wave duration (ms) and orientation (±) in leads V1 and V6.

T-wave assessment in the present study was based on waveform morphology (monophasic positive, monophasic negative, and biphasic) rather than amplitude. In contrast, Davignon et al. reported T-wave amplitudes without defining waveform morphology. Therefore, comparisons regarding T-wave findings are qualitative and descriptive in nature.

The P axis, T axis and QRS axis were calculated as the mean vector angle on the Einthoven plane, taking into account the amplitudes of these waves in the DI and aVF leads. A P-axis between 0° and 90° was considered normal sinus rhythm, whereas values outside this range were considered abnormal sinus rhythm. For the QRS axis, a range of 70° (10–125°) was considered normal, and values outside this minimum and maximum range were considered abnormal. A QRS axis between 125° and 180° was classified as right axis deviation (RAD), that between 0° and −90° was classified as left axis deviation (LAD), and that between −90° and −180° was classified as superior axis deviation. The QRS-T angle was defined as the absolute difference between the QRS axis and the T axis, with a difference >90° considered pathological [6]. The results were compared with the ECG data reported by Davignon et al. for descriptive and contextual purposes only, as their recordings were obtained between 0 and 1 day of age and cannot be considered direct normative reference values for the first postnatal hour [4].

In our study, ECG recordings were obtained after routine postnatal stabilization, with a median time of 25 min after birth (range: 10–60 min). All recordings were performed while the neonates were placed under an open warmer in the delivery room.

At the time of ECG acquisition, the majority of infants were in a calm or quietly awake state. Recordings were postponed in cases of excessive crying or agitation in order to minimize motion artifacts and physiologic stress.

Echocardiography was performed via a Philips Affiniti 50 ultrasound machine (Philips Affiniti 50 Cardiac Ultrasound, Bothell, WA, USA) equipped with a transducer operating at a carrier frequency of 4.2 or 8.3 MHz. Standard pediatric imaging windows were used, including parasternal (long- and short-axis), apical (four- and five-chamber), subcostal and suprasternal views. Morphological assessment was performed via the segmental approach, which assesses the direction of blood flow. The main components of this approach include the atrial situs, venoatrial connections (systemic and pulmonary venous return), atrioventricular (AV) connections, ventricles, ventriculo-arterial (VA) connections, spatial positions of the great arteries relative to each other, intracardiac defects, and extracardiac vascular anomalies. Congenital heart disease was classified according to definitions found in the literature [5]. Echocardiography was performed by the same pediatric cardiologist according to the outpatient clinic patient number. Echocardiographic examination was performed either on the same day or before discharge (on the 1st and 3rd days).

### Statistical Methods

The distribution of variables in the study was categorized via computer software, and descriptive results were obtained via SPSS version 23 (Statistical Package for the Social Sciences for Windows). Descriptive results are expressed as the mean ± standard deviation (Std), median (interquartile range), and percentage percentile. Chi-square tests and Fisher’s exact tests were used to compare categorical variables with normal normograms. *p* < 0.05 was considered statistically significant.

## 3. Results

There were 490 births during the study period, and 260 cases were included in the study. Among the patients, 50% were male (n = 130), with a mean gestational age of 38.1 ± 1.4 weeks and a mean birth weight of 3.2 ± 1.4 kg. The demographic data of the patients are shown in Table 1. As shown in Table 1, 6% of neonates were classified as SGA and 10% as LGA, which contributed to the relatively wide standard deviation despite a predominantly term cohort.

In our cohort, cesarean deliveries were predominantly performed under regional anesthesia (spinal or epidural), in accordance with routine obstetric practice at our institution, while general anesthesia was used in a limited number of cases.

A grade 1/6 systolic murmur was detected at the lower left sternal border in two neonates during cardiac examination.

The ECG findings and variations in the cases are shown in Table 2. In the ECG recordings, low atrial rhythm was detected in 0.3% of the patients (n = 1). Right axis deviation was observed in 1.5% of the patients (n = 4), and left axis deviation was observed in 1.2% of the patients (n = 3). A total of 0.3% of the patients (n = 1) had an abnormal P-axis, and 0.3% of the patients (n = 1) had an abnormal QRS-T-angle.

No statistically significant differences were found between the other parameters.

Echocardiography was performed during the first three days of life before discharge. Sixteen patients (6.1%) had patent foramen ovale in the interatrial septum, four patients (1.5%) had patent ductus atreiosus (hemodynamically insignificant), two patients (0.6%) had atrial septal defects (moderate, with 6 mm color Doppler flow), and one patient (0.3%) had ventricular septal defects (small midline, 2 mm width). In our cohort, no neonates were found to have hemodynamically significant ductal patency on subsequent echocardiographic evaluation. All identified patent ductus arteriosus cases were small and hemodynamically insignificant, and none required medical or interventional treatment.

## 4. Discussion

In this prospective observational study, we described electrocardiographic parameters recorded during the first postnatal hour in healthy term newborns. Our findings demonstrate that physiologic electrocardiographic patterns can be observed during this immediate transitional period.

ECG is a useful medical test for assessing the electrical activity of the heart and, to some extent, the structures of the heart. The primary parameters assessed via ECG include rhythm, rate, P axis, PR interval, QRS duration and axis, T wave characteristics, the QTc interval and ST-T changes, which are important in the diagnosis of various structural or congenital heart diseases [4,6,7]. Both term and preterm infants undergo cardiovascular changes at birth, coinciding with the transition from placental circulation to the lung as the respiratory organ. Changes in fetal cardiac physiology during late pregnancy and perinatal transition result in variability in neonatal ECG parameters [8]. In addition, studies focusing on neonatal ECG data are limited.

The most widely cited historical pediatric electrocardiographic reference dataset was reported by Davignon et al [4]. and was based on measurements from 2141 children. However, these recordings were obtained within the first 24 h of life and therefore do not specifically represent electrocardiographic findings during the first postnatal hour. Accordingly, Davignon’s data were considered as historical context rather than direct normative references for our study population [4].

In our study, heart rate, mean QRS axis, QRS duration, and R-wave and S-wave amplitudes in leads V1 and V6 measured in the first hour after birth were similar. The results were also consistent with those of recent studies [8,9].

A recent study revealed significant differences in the orientation of T waves in leads V1, V2, V3 and V4 across different age groups. The large proportion of positive factors in the neonatal group can be explained by the rapid pressure applied to the lungs, which directly affects the early phase of ventricular repolarization. As pulmonary pressure decreases in the first years of life, it has been suggested that infants may rely more on T-wave derivations (V1 and V4) to maintain repolarization [10]. Although the precise mechanism underlying this phenomenon remains unclear, it may be influenced by negative T V1 and various factors, including congenital heart disease, pulmonary arterial hypertension, myocardial ischemia, oxygen deficiency, and adrenaline levels [11].

In their study, Pimenta et al. [3] reported a significant difference in T wave positivity in leads V1–4 between neonates studied within the first 24 h and those studied between days 1–3 and 3–7. Similarly, in our study, we observed a predominance of positive T-wave morphology in lead V1, which is consistent with Pimenta’s findings. Additionally, Meng et al. [11] reported that T waves change 15 h after birth and that there is a relationship between PDA and pulmonary hypertension. In their cohort, the biphasic and positive T wave incidence was 85%, and the negative T wave incidence was 15%. Although they reported that a negative T wave after 15 h of birth was associated with a higher incidence of PDA, our findings did not support this finding. These findings may be related to the reduction in pulmonary vascular resistance during the first postnatal hour. Recognition of physiologic electrocardiographic patterns during the first postnatal hour may assist clinicians in distinguishing transitional findings from pathologic abnormalities.

Limitations: The main limitation of this study is that it was conducted in a single center with a limited number of patients. Owing to the challenges of obtaining ECG recordings in neonates, all recordings were performed by the same team. The inability to perform echocardiography immediately in the early period may have affected the results of the measurements, particularly the presence of patent ductus arteriosus and patent foramen ovale in the interatrial septum. Our number of Cesarean section patients is relatively high because our hospital is a referral center for gynecology and obstetrics, and the drugs that are used for anesthesia might affect ECGs. In addition, as the results were compared with those of Davignon and colleagues, racial and ethnic factors may have influenced the results.

## 5. Conclusions

In conclusion, physiologic ECG differences can be observed in neonates during the first postnatal hour. In particular, T wave positivity was significantly greater in the first hour after birth. Therefore, studies with larger numbers of cases are needed for a clearer interpretation of ECG findings in this age group.

## Figures and Tables

**Table 1:** General Characteristics of the Cases.

Variable	n = 260
Mother’s Age, years	27 ± 3
Gestational Age, weeks	38.1 ± 1.4
Delivery Type,	
NSD (Normal Spontaneous Delivery)	100 (38)
CS (Caesarean Section)	160 (62)
Birth Weight, kg	3.2 ± 1.4
Classification	
Small for Gestational Age	16 (6)
Appropriate for Gestational Age	218 (84)
Large for Gestational Age	26 (10)
Gender, Male	130 (50)
Ethnicity, (Turkish, %)	214 (82)
Maternal Drug Use	
Levothrone	2 (0.7)
Alpha methyl dopa	1 (0.3)
Low dose aspirin	2 (0.7)
Novorapid	1 (0.3)
Methimazole	1 (0.3)

n (%) or mean ± standard derivation.

**Table 2:** Electrocardiographic Parameters in the Study Population.

Variable	
Heart rate, bpm	120 (110–130)
Amplitude P Lead II (mv)	1 (0.7–1.4)
PR Lead II (msec)	90 (80–100)
QT Lead II (msec)	310 (290–340)
QTc (msec) (Bazzett)	400 (360–440)
QRS axis (°)	130 (100–160)
Amplitude Q DIII (mV)	0.40 (0.20–0.60)
Amplitude Q V6 (mV)	0.10 (0.06–0.15)
Amplitude R V1 (mV)	1.25 (1.10–1.50)
Amplitude R V2 (mV)	1.40 (1.00–1.80)
Amplitude R V5 (mV)	1.40 (1.00–1.60)
Amplitude R V6 (mV)	1.30 (0.90–1.70)
Amplitude S V1 (mV)	1.00 (0.60–1.40)
Amplitude S V2 (mV)	1.55 (1.20–1.90)
Amplitude S V5 (mV)	0.90 (0.50–1.50)
Amplitude S V6 (mV)	0.60 (0.40–0.80)
R/S V1	1.4 (1.0–1.8)
R/S V6	5.2 (3.0–8.0)
Duration QRS Lead II (msec)	45 (40–50)
V1 T	
Positive	130 (50)
Negative	65 (25)
Biphasic	65 (25)
V6 T	
Positive	234 (90)
Negative	26 (10)
Biphasic	-
QRS axis	
Normal	253 (97.3)
Right Axis	4 (1.5)
Left Axis	3 (1.2)
Abnormal QRS-T angle	1 (0.3)
Rhythm	
Normal	259 (99.7)
Abnormal	1 (0.3)

n (%) or median (range).

## Data Availability

All the data generated or analyzed during this study are included in this article. Further inquiries can be directed to the corresponding author.

## Abbreviations

RV	right ventricle.
LV	left ventricle.
ECG	electrocardiography.
RAD	right axis deviation.
LAD	left axis deviation.
AV	atrioventricular.
VA	ventriculo-arterial.
PDA	patent ductus arteriosus.
